# Harnessing Neutrophil Survival Mechanisms during Chronic Infection by *Pseudomonas aeruginosa*: Novel Therapeutic Targets to Dampen Inflammation in Cystic Fibrosis

**DOI:** 10.3389/fcimb.2017.00243

**Published:** 2017-06-30

**Authors:** Benoît S. Marteyn, Pierre-Régis Burgel, Laurent Meijer, Véronique Witko-Sarsat

**Affiliations:** ^1^Unité de Pathogénie Microbienne Moléculaire, Institut PasteurParis, France; ^2^Institut National de la Santé et de la Recherche Médicale, U12021202Paris, France; ^3^Institut Gustave RoussyVillejuif, France; ^4^Université Paris Descartes, Sorbonne Paris CitéParis, France; ^5^Pneumology Department, Hôpital CochinParis, France; ^6^ManRos Therapeutics, Centre de PerharidyRoscoff, France; ^7^Institut National de la Santé et de la Recherche Médicale, U1016, Institut CochinParis, France; ^8^Centre National de la Recherche Scientifique-UMR 8104Paris, France; ^9^Center of Excellence, Labex InflamexParis, France

**Keywords:** inflammation, cystic fibrosis, Pseudomonas, PCNA, apoptosis, hypoxia, roscovitine

## Abstract

More than two decades after cloning the cystic fibrosis transmembrane regulator (CFTR) gene, the defective gene in cystic fibrosis (CF), we still do not understand how dysfunction of this ion channel causes lung disease and the tremendous neutrophil burden which persists within the airways; nor why chronic colonization by *Pseudomonas aeruginosa* develops in CF patients who are thought to be immunocompetent. It appears that the microenvironment within the lung of CF patients provides favorable conditions for both *P. aeruginosa* colonization and neutrophil survival. In this context, the ability of bacteria to induce hypoxia, which in turn affects neutrophil survival is an additional level of complexity that needs to be accounted for when controlling neutrophil fate in CF. Recent studies have underscored the importance of neutrophils in innate immunity and their functions appear to extend far beyond their well-described role in antibacterial defense. Perhaps a disturbance in neutrophil reprogramming during the course of an infection severely modulates the inflammatory response in CF. Furthermore there is an emerging concept that the CFTR itself may be an immune modulator and stimulating CFTR function in CF patients could promote neutrophil and macrophages antimicrobial function. Fostering the resolution of inflammation by favoring neutrophil apoptosis could preserve their microbicidal activities but decrease their proinflammatory potential. In this context, triggering neutrophil apoptosis with roscovitine may be a potential therapeutic option and this is currently being evaluated in CF patients. In the present review we discuss how neutrophils functions are disturbed in CF and how this may relate to chronic infection with *P. aeuginosa* and we propose novel research directions aimed at modulating neutrophil survival, dampening lung inflammation and ultimately leading to an amelioration of the lung disease.

## Chronic infection and inflammation in cystic fibrosis are indicative of a defect in the immune response

**Figure 1:**
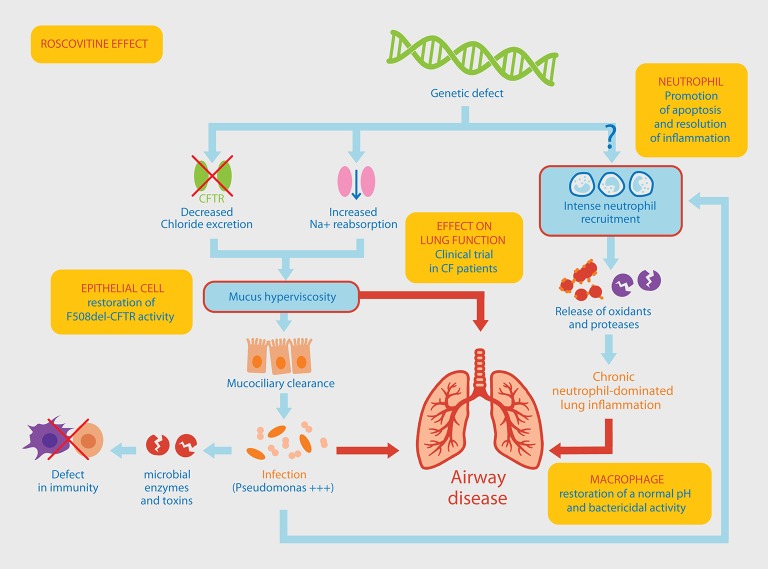
Pathophysiological mechanisms involved in *Pseudomonas aeruginosa* chronic lung infection associated with neutrophil-dominated inflammation. The original genetic defect in CF mutations within the *CFTR* gene leading to a defective chloride ions excretion and an increased sodium reabsorption, resulting in the production of a thick mucus in the airways. Altered mucociliary clearance favors an increased susceptibility to infections, a chronic colonization of the lung by *P. aeruginosa*, a massive neutrophil recruitment within the airways and a persistent airway inflammation. An alternative pathway to this classical view of the CF could be a “constitutive defect in innate immunity” linking the genetic defect to the intense recruitment of neutrophils. This would result in an increased activation state (release of oxidants and proteases) and a delayed apoptosis explaining their persistence at the site of inflammation. Yellow boxes indicate the different effects of roscovitine (see main text for details) which is currently tested in a CF clinical trial.

Cystic fibrosis (CF) is the most frequent hereditary genetic disease in the Caucasian population, originating from mutations within the Cystic Fibrosis Transmembrane Conductance Regulator (CFTR) gene. These mutations result in an often-lethal respiratory disease affecting approximately 1 in 4,500 births in Europe and North America. Median predicted survival at birth ranges from 40 to 50 years in developed countries and longevity continues to improve (MacKenzie et al., 2014), with a predicted increase in the number of CF patients (Burgel et al., 2015). Mutation of the CFTR leads to the secretion of a viscous and abundant mucus in the lung that is conducive to bacterial infections (Martin et al., 2014; Figure 1). In fact, almost all patients suffer from pulmonary infections caused by various pathogens, which can be isolated from sputum or bronchoalveolar lavages. Infections are treated by administrating antibiotics and in the long term this generates bacterial multi-resistance. Pulmonary bacterial infections are the major cause of death (Martin et al., 2016a).

Neutrophils are the first and the most abundant leukocyte that migrate to the site of infection followed by monocytes, which differentiate locally into macrophages. In the acute phase of infections or inflammatory diseases, neutrophils can engulf and kill invading microorganisms (Witko-Sarsat et al., 2000). In CF, several studies have shown abnormalities in innate immunity where neutrophils are major instigators (Bals et al., 1999; Cohen and Prince, 2012; Bonfield, 2015). Neutrophils can no longer migrate through viscous mucus, thereby reducing the capture and destruction of bacteria (Matsui et al., 2005). Chronic pulmonary neutrophil-dominated inflammation occurs very early in CF patient and it represents a key element in disease severity (Cantin, 1995; Khan et al., 1995). The underlying mechanisms contributing to the disease remain largely unknown, raising the question of a relationship between genetic deficit, chronic pulmonary inflammation and specificity of the infection by *P. aeruginosa* (Hayes et al., 2011; Figure 1).

CF airways contain an abundant amount of nuclear material that is believed to originate from neutrophils. This pathophysiological characteristic is linked to the ability of neutrophils to release DNA through neutrophil extracellular traps (NETs), something which has been described in CF (Manzenreiter et al., 2012; Dwyer et al., 2014). Targeting nuclear material with DNase I improved lung function, putatively through NETs degradation (Gray et al., 2015). NETs can kill *P. aeruginosa*, although acquired resistances have been described among clinical isolates (Young et al., 2011). Oxidative burst and NADPH activity are essential to the process of NET formation with myeloperoxidase (MPO) and neutrophil elastase (NE), which stand as essential co-factors (Papayannopoulos et al., 2010). The main issue in the characterization of NETs in CF is to decipher the extent to which the abundant nuclear material accumulation in CF airways originate from dead cells or if, as proposed, it is the result of an active and coordinated process of release through NETs. Further investigations will be required to address this controversial question.

## *Pseudomonas aeruginosa*: a well-adapted bacteria to the CF lung

While there is not usually any pathogenic microbe in respiratory secretion in CF patients at birth, systematic bronchoalveolar lavage of young children with CF reveals that bacterial infection usually occurs within the first months of life and is associated with lung function impairment and airway structural damage (Ramsey et al., 2014). The nature of the microbes infecting CF lungs depend on the patient and varies with age: the predominant bacteria during childhood are usually *Staphylococcus aureus* and *Haemophilus influenzae*, whereas *P. aeruginosa* is the major pathogen cultured from CF airways in adolescents and adults (Vaincre la Mucoviscidose et Ined)^1^. One of the first microbes identified in the CF airways is *S. aureus*, a gram positive bacterium. It has been suggested that early *S. aureus* infection, which contributes to structural damage of the airways, as well as antibiotic treatments, promote the implantation of *P. aeruginosa* (Govan and Nelson, 1993; Ratjen et al., 2001). The first positive culture containing *P. aeruginosa* can occur either within the first few months of life or are delayed by several decades (Pernet et al., 2014). For example, *P. aeruginosa* infection is less prevalent in patients with CFTR mutations associated with residual CFTR function (Burgel et al., 2010) and variants in dynactin 4 have been associated with late occurrence of the first *P. aeruginosa* culture in CF patients (Emond et al., 2012; Viel et al., 2016).

Chronic *P. aeruginosa* infection is responsible for increased morbidity and mortality (Emerson et al., 2002). Recent studies have shown that the predominance of *P. aeruginosa* in older CF patients was not due solely to a resistance to antibiotics. Indeed, the bacterium itself may manipulate host immune responses by stimulating airway epithelium cells to produce phospholipase A2 type-IIA (sPLA2-IIA) which is capable of killing Gram positive bacteria such as *S. aureus* (Pernet et al., 2014).

## Hypoxic conditions in CF and the impact on *P. aeruginosa* virulence and immune response efficiency

Lungs, along with the skin, are the human organs exposed to the highest oxygen levels, due to the direct contact between the epithelium and atmospheric air. The alveolar partial pressure of oxygen (pO_2_) is 100–110 mmHg (vs. 160 mmHg in atmospheric air; Pittman, 2013). Oxygen diffuses through a thin mucus layer (2–10 μm in trachea), mainly composed of Muc5ac and Muc5b gel-forming mucins (Burgel et al., 2007). It has been reported that hypoxia is induced in the airways of CF patients (Worlitzsch et al., 2002). Similar observations were made during other pathogen infections including *Shigella flexneri* (Arena et al., 2017) or *Mycobacterium tuberculosis* (Tsai et al., 2006; Rustad et al., 2009) and in sterile inflammations (Karhausen et al., 2004; Campbell et al., 2014). More specifically, it has been shown that induction of hypoxia is associated with the establishment of an oxygen gradient within a thicker mucus layer in CF patients where *P. aeruginosa* was preferentially found within the hypoxic niches (Worlitzsch et al., 2002). Although reactive oxygen species (ROS) production has been shown *in vitro* to increase mucin expression (Muc5ac; Yan et al., 2008), no clear link has been defined between the induction of hypoxia and mucin production in CF patients. However, the increased production of mucins in CF lung disease is anticipated to exacerbate hypoxia.

*P. aeruginosa* is a Gram-negative, rod-shaped facultative anaerobe, which has the ability to colonize a wide range of microenvironments. *P. aeruginosa* aerobic respiration relies on the reduction of O_2_ which is mediated by Cytochrome *cbb3* oxidase, Cytochrome *aa3* oxidase and Cytochrome *bo3* oxidase. Alternatively, in the absence of available oxygen, *P. aeruginosa* energy production is mediated by the anaerobic respiratory chain allowing for the reduction of nitrate (nitrate reductase) or nitrite (nitrite reductase) (Cook et al., 2014). This dual respiratory capacity appears to be essential to allow *P. aeruginosa* to colonize lung mucus and to persist within the hypoxic environment. The metabolic shift associated to *P. aeruginosa* adaptation under hypoxic conditions was recently studied: a set of genes specifically expressed under hypoxic conditions were identified (*azu, cbb3-1, cbb3-2, ccpR, icd, idh, oprF, himD*, and *nuoA*) and these genes were proposed to stand as markers for hypoxic adaptation of *P. aeruginosa* within the CF lung environment (Eichner et al., 2014).

Two hypotheses have been proposed to explain hypoxia induction within CF mucus layer. Either *P. aeruginosa* is responsible for oxygen depletion or activated neutrophils consume dioxygen for reactive oxygen species production catalyzed by NADPH oxidase, as reported during sterile inflammation (Campbell et al., 2014). Neutrophil NADPH oxidase function has been shown to play an essential role in *P. aeruginosa* killing (Mizgerd and Brain, 1995). The impact of hypoxia on the propagation of infection is currently unclear: does it promote *P. aeruginosa* colonization capacity or does it foster the immune response efficiency? To date this question remains unanswered.

The impact of hypoxia on CF is not yet fully understood and has been controversial to some extent. It has been shown that hypoxia protects epithelial cells from *P. aeruginosa* internalization *in vitro*, suggesting that mimicking hypoxia responses *in vivo* with hydroxylase inhibitor dimethyloxallyl glycine (DMOG) could represent a potential therapeutic approach (Schaible et al., 2012, 2013). Conversely, it has been reported that oxygen-limiting conditions increase antibiotic tolerance, biofilm formation, and alginate synthesis, promoting *P. aeruginosa* persistence (Schobert and Tielen, 2010). Induction of hypoxia would promote neutrophils survival, as these cells are mostly glycolytic, producing ATP mainly through glycolysis not respiration (Maianski et al., 2004). Neutrophils are well adapted to hypoxia and this is mostly attributed to the transcriptional regulator HIFs (1 and 2; Walmsley et al., 2005; Monceaux et al., 2016).

NETs have been observed in CF airways and characterized as the most efficient neutrophil antimicrobial function. However, their antimicrobial function together with the associated cell-death process (named NETosis) have been challenged by several authors (Simon et al., 2013; Malachowa et al., 2016; Nauseef and Kubes, 2016). Here, we focus on the relevance of NETs formation and antimicrobial function in the context of hypoxic microenvironments. Dioxygen is a unique substrate of the NADPH oxidase, which catalyze singlet oxygen production (O2^•−^). In hypoxic environments, NADPH oxidase activity and ${\text{O}}_{2}^{•-}$ production are likely limited. NADPH oxidase activity has been shown to be essential for NETs formation (Kirchner et al., 2012; Palmer et al., 2012), similarly to ROS, including hydrogen peroxide (H_2_O_2_) (Fuchs et al., 2007) or singlet oxygen (Nishinaka et al., 2011). Under hypoxic conditions, NETs production may be decreased, promoting *P. aeruginosa* persistence in these microenvironments. Further investigations will be required to address this important question, as most studies were performed *in vitro* under atmospheric conditions, which do not reflect pathophysiological conditions.

Hypoxia induction should be seriously considered to fully comprehend CF causes and clinical outcomes and also in the development of novel antimicrobial molecules targeting *P. aeruginosa*. It was recently reported that hypoxia promoted *P. aeruginosa* antibiotic resistance by modulating multidrug efflux pumps composition and subunits stoichiometry (Eichner et al., 2014). Deciphering the extent to which hypoxia modulates *P. aeruginosa* virulence mechanisms and impacts on its adaptation within infection foci requires further investigations in CF animal models.

## Neutrophil functions in CF: still under investigation

Dissecting effector functions in CF neutrophils, already detailed in extensive reviews (Downey et al., 2009; Hayes et al., 2011; Laval et al., 2016), has not revealed a major deficiency in neutrophil functions. However, it has been described that airway neutrophils exhibit profound functional (Laval et al., 2013) and signaling changes mediated by the cytoskeleton-associated kinase that regulate granule exocytosis (Tirouvanziam et al., 2008). Likewise, microarray analysis was used to compare the expression of more than 1,000 genes in neutrophils isolated from blood of CF patients and healthy donors and it revealed the upregulation of 62 genes including those encoding chemokines, and downregulation of 27 genes suggesting a specific disturbance in the mechanisms regulating inflammation (Adib-Conquy et al., 2008).

Although the presence of CFTR in neutrophils was controversial for years, it has been detected in membranes (Pohl et al., 2014) and its localization in both secretory vesicles and phagolysosomes has been reported (Painter et al., 2006). Upon neutrophil activation, NADPH assembly combined with myeloperoxidase chlorinating activity leads to the generation of oxidants essential for bacterial killing (Nauseef, 2007). CF neutrophils exhibit a defective intraphagolysosomal HOCl production, although a normal extracellular production of MPO-derived HOCl is observed (Painter et al., 2006). Chloride is essential for *P. aeruginosa* killing by neutrophils. CF neutrophils have a reduced bactericidal capacity compared non-CF neutrophils in the presence of chloride, strongly suggesting that a defective CFTR might compromise the ability of CF neutrophils to clear *P. aeruginosa* (Painter et al., 2008). These results are consistent with previous reports regarding the role of CFTR in the acidification of macrophages' phagolysosome (Di et al., 2006). Accordingly, CFTR activation represents an appealing therapeutic strategy and this is currently in development (Son et al., 2017).

## Defect in neutrophil apoptosis in CF leading to failure in the resolution of inflammation

Neutrophil apoptosis, a process of programmed cell death that prevents the release of neutrophil histotoxic contents including oxidants and proteinases, is tightly regulated and limits the destruction of surrounding tissue (Geering and Simon, 2011; Witko-Sarsat et al., 2011). The subsequent recognition and phagocytosis of apoptotic neutrophils by macrophages is central to the successful resolution of an inflammatory response (Kennedy and DeLeo, 2009) and to avoid autoimmunity (Thieblemont et al., 2016). Dying neutrophils exert an anti-inflammatory effect through modulation of macrophage inflammatory cytokine release (Bratton and Henson, 2011). Neutrophil apoptosis may be delayed, induced or enhanced by micro-organisms depending on their immune evasion strategies and the health of the host they encounter (McCracken and Allen, 2014).

Several abnormalities have been described in macrophages from CF patients but so far no defect in the phagocytosis of apoptotic cells has been reported (Bruscia and Bonfield, 2016). This was illustrated in macrophages from CFTR−/− mice infected with *P. aeruginosa* showing an enhanced cytokine production and secretion suggesting that the macrophage response may be an important therapeutic target for decreasing the morbidity of CF lung disease (Bruscia et al., 2009). At the site of infection, neutrophils represent more than 95% of the cells from the bronchoalveolar lavage and increased neutrophil lifespan is critical for effective host defense but delays in apoptosis can lead to persistent tissue damage. In neutrophils, analysis of genes expression during inflammation clearly showed a modulation of genes involved in apoptosis (Kobayashi et al., 2003).

The short-lived pro-survival Bcl-2 family protein, Mcl-1 (myeloid cell leukemia-1), is instrumental in controlling apoptosis in response to inflammatory stimuli (Moulding et al., 2001; Milot and Filep, 2011). Notably, neutrophils from CF patients chronically infected with *P. aeruginosa* have a prolonged survival (Dibbert et al., 1999; McKeon et al., 2008). An increased survival was also found in neutrophils isolated from the parents of CF patients, suggesting that at least in part, the defect in neutrophils may be genetically determined (Moriceau et al., 2010). In the latter study, the delayed apoptosis observed in CF patients was not reversed by inhibition of CFTR functions strongly suggesting that some CFTR functions in neutrophils may be independent of its chloride channel and rather due to its ability to associate with other proteins regulating cell functions.

## Promoting resolution of inflammation by targeting neutrophils: the case of roscovitine

Today, treatment of inflammatory diseases with non-steroidal anti-inflammatories is based on inhibiting the synthesis or action of inflammatory mediators that drive the host response to injury (Perretti et al., 2015). An alternative approach for the development of novel therapeutics is now based on endogenous mechanisms that switch off acute inflammation and bring about its resolution (Serhan et al., 2007). To date, conventional anti-inflammatory therapies in CF, using glucocorticoids or non-steroid anti-inflammatory drugs, such as ibuprofen, have shown beneficial, albeit marginal, effects by slowing down CF disease progression (Eigen et al., 1995). These modest results may be attributed to the fact that none of these treatments specifically targeted neutrophils, which represent the main cellular actors in inflammation associated with lung disease in CF. In addition, serious side effect of prednisone on growth in children have precluded their therapeutic use in CF (Lai et al., 2000). Inhibition of neutrophil elastase has been tested in order to decrease lung inflammation and is still under investigation (Kelly et al., 2008). A strategy based on interfering with neutrophil recruitment using BIIL 284, an LTB4 receptor (Konstan et al., 2014) or SB 656933, a CXCR2 (Moss et al., 2013) antagonist have been investigated in CF. Unfortunately, these compounds appear to enhance inflammation and have proven to be detrimental to the clinical status of the CF patients. Consequently, novel approaches need to regulate rather than inhibit neutrophils in CF.

In line with this, neutrophil apoptosis represents an important mechanism in the resolution of inflammation and could be considered as a good target to dampen inflammation in CF (Jones et al., 2016). Roscovitine is a low molecular weight pharmacological inhibitor of cyclin-dependent kinases (CDKs) discovered over 20 years ago during studies on the regulation of cell division in starfish oocytes (Meijer et al., 1997; Meijer and Raymond, 2003). This molecule has been used as a pharmacological tool to investigate cell cycle control, apoptosis, neuronal functions, etc. Furthermore roscovitine has been evaluated as a drug candidate in numerous diseases ranging from cancers, especially neuroblastoma (Bettayeb et al., 2010; Delehouze et al., 2014), viral infections, neurodegeneration, rheumatoid arthritis, glaucoma to polycystic kidney disease. Roscovitine has already been administrated to over 500 patients. While roscovitine was originally believed to exert its effects mainly on proliferating cells, it was reported that roscovitine also affected neutrophils which are deficient in proliferative capacities (Savio et al., 2006; Leitch et al., 2009). In neutrophils, roscovitine triggers apoptosis thereby favoring their phagocytosis by macrophages to promote the resolution of inflammation (Rossi et al., 2006). Notably, this activity was due to the inhibition of CDK7 and CDK9 involved in the regulation of RNA transcription (Leitch et al., 2012). Roscovitine has proven beneficial in enhancing neutrophil apoptosis in a model of meningitis (Koedel et al., 2009). However, the modulation of innate and adaptive immunity of roscovitine extends beyond its effect on neutrophils (Meijer et al., 2016). Roscovitine can act on CF alveolar macrophages to rescue acidification in phagolysosomes, which show abnormally high pH (Di et al., 2006). As a result, roscovitine restores their bactericidal activity (Riazanski et al., 2015). Whether roscovitine can modulate the microbicidal activities of neutrophils from healthy subjects or from CF patients is yet to be tested and should be addressed. In addition, roscovitine can correct the CFTR defect, as it partially protects F508del-CFTR from proteolytic degradation and favors its trafficking to the plasma membrane (Norez et al., 2014). Altogether, roscovitine has multiple activities resulting in a strong therapeutic potential in CF and this is currently being evaluated in a first clinical trial with *P. aeruginosa* infected CF patients (Meijer et al., 2016; Figure 1).

However, the question remains whether enhancing neutrophil clearance could represent a potential danger of decreasing the antibacterial defense provided by neutrophils. Importantly, CF patients are not prone to neutropenia (the definition of which is an absolute blood count of 500/mm^3^ in non-CF individuals, Bodey et al., 1966) and the only cases reported in CF so far were drug-induced.

## PCNA scaffold as a novel key to favor neutrophil apoptosis: regulatory role of p21/waf1 in lung inflammation during persistent *P. aeruginosa* infection

Neutrophils are terminally differentiated cells deprived of proliferating capacities, and are committed to death. Despite their lack of proliferation, we have discovered that mature neutrophils express high levels of proliferating cell nuclear antigen (PCNA), which was exclusively localized in the cytosol (Witko-Sarsat et al., 2010). This was unexpected because PCNA was known a nuclear factor involved in DNA replication and repair of proliferating cells (Moldovan et al., 2007), Notably, in mature neutrophils PCNA plays a pivotal role in neutrophil survival and changes in parallel with neutrophil apoptosis (Witko-Sarsat and Ohayon, 2016). In the neutrophil cytosol, PCNA was associated with procaspase-8 and procaspase-9 to prevent their activation. In accordance with our hypothesis that PCNA promotes neutrophil survival, we previously showed by immunocytochemistry that PCNA is highly expressed in inflammatory neutrophils within CF lung explants (Chiara et al., 2012). In keeping with these latter results, PCNA is more abundant in the cytosol of CF neutrophils (Western blot analysis) compared to non-CF neutrophils (Martin et al., 2016b). We have provided evidence that treatment with the p21 peptide (Warbrick, 2000), capable of binding the PCNA interdomain connecting loop, reversed the delay in apoptosis observed in neutrophils from CF patients and restored apoptosis levels to that of healthy controls (Martin et al., 2016b). Hence, the pro-apoptotic effect of the p21 peptide is a proof-of-concept that p21/waf1 interfers with cytoplasmic PCNA to trigger neutrophil apoptosis (Chiara et al., 2012). However, p21 protein is hardly expressed in neutrophils and it is unlikely that endogenous p21 regulates neutrophil apoptosis under basal state. In sharp contrast, p21/waf1 mRNA was strongly induced in human neutrophils following LPS challenge suggesting that p21/waf 1 was involved in the regulation of neutrophil activation under inflammatory conditions (Martin et al., 2016a).

To understand the potential role of p21/waf1 in *P. aeruginosa* infection in the lung, we used a model of persistent lung infection triggered by the instillation of agarose beads-coated *P. aeruginosa* in mice which results in accumulation of neutrophils in peribronchial area and in alveolar consolidation (Martin et al., 2016b). After 7 days of lung infection with *P. aeruginosa*, inflammation was more intense in p21^−/−^ mice compared to WT as evidenced by morphologic analysis of the lung. Since no intrinsic defect in the phagocytosis of apoptotic neutrophils by macrophages is found in p21^−/−^ mice, the accumulation of neutrophils at the site of inflammation in these mice could be attributable to a defect in neutrophil apoptosis rather than impaired clearance by macrophages. Accordingly, *in vitro*, neutrophils isolated from p21^−/−^ mice displayed enhanced survival in response to TNF-α and G-CSF. In keeping with these data obtained *in vivo* in murine models, an induction of p21 mRNA was observed in responses to both cytokines in human neutrophils (Martin et al., 2016b). Our study provides clear evidence that p21/waf1 expression is a key regulator of neutrophil fate *in vivo*, especially during *P. aeruginosa* infection. In conclusion, similarly to roscovitine, targeting PCNA in neutrophils using the p21 competing peptide could accelerate the resolution of inflammation in an infectious context and could be considered as a potential therapeutic strategy in CF.

## Remaining open questions: the dilemma of targeting neutrophil survival in CF

We urgently need to identify the molecular mechanisms underlying neutrophil dysfunction in CF, how it relates to CFTR and how it promotes infection with *P. aeruginosa*. Given the importance and the renewed interest in neutrophils as instrumental actors in immune deregulation associated with lung disease in CF, promoting CFTR-dependent antimicrobial function (Son et al., 2017) or targeting neutrophils to promote their apoptosis (Martin et al., 2016b) is a timely issue that should be addressed. The persistence of neutrophils in CF airways relies on multiple parameters and this enigma will be solved by taking into account the complexity of neutrophil plasticity in response to the hypoxic inflammatory microenvironment and the influence of *P. aeruginosa* on neutrophil survival mechanisms.

## Author contributions

All authors have written, discussed and approved the final manuscript. VW is an expert in neutrophil-Pseudomonas aeruginosa interaction expert in CF. BM is an expert in bacteria infection and hypoxia. PB is a medical doctor involved in CF patient care. LM is an expert in roscovitine treatment.

### Conflict of interest statement

Laurent MEIJER is CEO & CSO of ManRos Therapeutics. The other authors have no conflict of interest to declare.

## Footnotes

^1^Vaincre la Mucoviscidose et Ined Registre Français de la Mucoviscidose–Bilan des données 2015 [Online]. Paris. Available: (Accessed January 28th 2017).
